# A Case of Chlorosis, Accompanied by Obstruction of the Bowels, Which Continued Thirty-seven Days

**Published:** 1852-02

**Authors:** P. S. Shields

**Affiliations:** New Albany, Ia.


Art. II.—*A Case of Chlorosis, accompanied by Obstruction of the Bowels, which continued thirty-seven days.* By Dr. P. S. Shields, of New Albany, Ia.

The following case of *Chlorosis*, will probably be interesting to the medical profession from the anomalous symptoms developed during its progress. The subject was a young lady, Miss R——, aged eighteen years, of a rather tall, slender form; dark eyes and hair, and fair skin. Her habits were rather sedentary. She was at the time her health failed, pursuing a course of study in a Female Seminary in this city, and applied herself diligently to her studies, continuing them to late hours at night. Her catamenia from their first appearance have been either irregular in time, or deficient in quantity; consequently her health has not been good. The last appearance of her menses was during the time of her examination in July last, prior to graduation. From this time her health continued gradually to decline. Her lips and cheeks soon became exsanguinous; her eyes lost their lustre; skin became pale, cool, and dry; her pulse grew more frequent and feeble; her extremities cool; bowels torpid; appetite defective; she was troubled by a sense of stricture and oppression in the chest, and a pain in the region of the heart, accompanied by frequent attacks of palpitations.—Yet auscultation revealed nothing of organic derangement in this organ. She frequently suffers from pain or giddiness of the head, and indistinct vision; but there was no other cerebral disturbance. There was also occasionally pain across the loins. Her urine was generally limpid and more copious than natural, and was often tinged by the coloring matter of the articles of medicine she was taking. Sometimes it was turbid. About the 15th of September, a blue spot, of a crescent shape, two and a half inches in length and one in breadth, appeared under each eye; which in a day or two became of a dark indigo blue in the centre, fading towards the circumference into a light sky blue. They were seated immediately on the malar bones, extending from the nose, beyond the external canthus of the eye, leaving the eye-lids and cellular texture beneath of the natural hue. They had the appearance of having been painted, and gave to the physiognomy a most singular appearance. They continued permanent for nearly three months, occasionally a little darker at one time than another. On the 19th of October, she ceased to have any further action of the bowels. The food taken was retained one or two days in the stomach and then thrown up, either by vomiting or eructation, generally almost unchanged, and very seldom having the least taste or smell of acidity. There was no sensation of pain or motion in the abdomen, and no unusual fullness, hardness, or contraction of the abdominal muscles. Dr. H. M. Dowling, the attending physician, was called in about this time, and directed his treatment principally to the removal of the constipation of the bowels, and during the next fifteen days gave sixty grains of calomel divided in three different doses, with the same quantity of pulv. jalap., and senna and sulph. magnes., castor oil and aloetic pills, in large quantities. The most of these medicines were retained, and were followed at different times by various cathartic enemata.

Oct. 25th.—I saw the case for the first time in consultation with Dr. Dowling. We now determined to try the effect of distending the colon by injecting large quantities of warm water with spirits of turpentine. A flexible tube was accordingly introduced 10 or 12 inches up the rectum, and from two to three quarts of warm water, with two table-spoonfuls of spirits turpentine were thrown up once each day, and smaller saline enemata at other times during the day. They were generally retained from twenty to thirty minutes, when they came away unmixed with any fecal matter; except on one occasion, when a very small amount was passed, in size about as large as a hazle-nut, perfectly natural in color and consistency.

Nov. 6th.—Symptoms unchanged, and no alvine evacuation. We now prescribed one drop of croton oil in pill, with sapo. hisp., every four hours. The stimulating enemata to be continued.

8th.—She now retains her food well, and has taken and retained ten drops of an excellent article of croton oil; but still has had no alvine dejection, nor felt any pain, uneasiness, or sensation of motion in the abdomen.—Prescribed blue mass 3 grains, morning, noon and night; as also, ℞ tinct. aloes ℥ii, tinct. cardamom., ℥i; mix, and take ℥i morning and evening.

10th.—No change in the symptoms. Medicine continued. Urged to take as much nourishment as the stomach will retain. We now resorted to electricity in the form of Morehead's graduating galvanic battery. The current of electricity was sent in all directions through the abdomen and pelvis, and continued for fifteen or twenty minutes, morning and evening of each day.

15th.—During the last twenty-four hours, she has felt a sensation of motion in the abdomen, as if the intestines were slightly distended by flatus. The sensation is more pleasurable than painful; and there is rather more fullness of the abdomen. Her appetite and strength are improving; pulse fuller, and skin warmer. The same treatment continued.

16th.—Symptoms the same as yesterday.

17th.—She had, this morning, a free fecal evacuation, of a very natural color and consistency; and presenting no appearance of having been long retained, or of there being any unusual accumulation of it, and unaccompanied by the least pain or uneasy sensation. The same treatment was continued, only that the electricity was used once each day, and two grains of the hydrogenated ferruginous preparation of M. Quesneville were given, morning, noon, and night.

24th.—Her general health is much improved; less anemia; skin of natural temperature; pulse more full, and less frequent; regular daily fecal evacuations, of a healthy color and consistency; and appetite and strength improving. Electricity and blue mass discontinued. She has now taken, in the course of treatment, sixty grains of calomel, and about the same quantity of blue mass, without being mercurialized in the slightest degree. Continued the iron and tinctures.

Dec. 5th.—General health so much improved that she does not need to lie down during the day; but she is much depressed in spirits, from an apprehension of the permanent continuance of the blue spots under her eyes.—Continued the iron and the tinct. aloes and cardamom.—Prescribed unguent. iod. potas. by friction to the blue spots, morning and evening.

12th.—Patient much elated at the rapid and entire disappearance of the discoloration under the eyes, which took place to a great extent in 24 hours after the first application of the ointment. The general treatment to be continued.

23d.—Her health is about the same, with the exception of a frequent irritable cough, without expectoration, which is more frequent at night. Since the last report, the blue discoloration beneath the eyes reappeared slightly, but was immediately removed by the application of the iodine ointment. Prescribed for the cough the following: ℞. Acid. hydrocyan. gt. 32, syr. tolut. ʒii, aq. destil. ℥ii; mix, and take ʒss. every four hours.

Jan. 3d.—Cough relieved, and general health again improving. Electricity resumed.

Jan. 10th.—Her health is now as good as it has been at any time during the last two years. The precordial symptoms have entirely disappeared, but up to this time there has been no return of the menses. Electricity omitted; the iron, with the tinct. aloes and cardamom to be continued in conjunction with the daily use of injections of a dilute solution of aq. ammoniæ.

12th.—She left the city this morning on a visit to her friends. The injections are consequently omitted, and the tinct. guiacum of Dewees prescribed.

*Remarks.*—1st. The long continued concentration of the intellectual powers upon her studies, and the consequent neglect of exercise, and other physical means of promoting health, was clearly the cause of the chlorosis, and of the suppression of the menses, which latter was a consequence and not the cause of the disease. This conclusion is fully sustained by the great improvement in her general health, without the return of her catamenia.

2d. The obstruction of the bowels, was caused by defective innervation, producing almost complete paralysis of the intestinal canal. The correctness of this opinion is rendered probable by the complete success of electricity and tonics, after the most active cathartics had failed to produce intestinal action; and further, by the entire absence of pain or uneasiness in the abdomen under the use of the latter.

3d. The insusceptibility of the system to the specific effects of mercury doubtless resulted from the same cause; it was not absorbed and carried into the general system.

4th. The kidneys probably performed a vicarious function, in this case, eliminating matters, which should have been evacuated by the bowels, and preventing any serious accumulation of feculent matter in the large intestines.

5th. The discoloration beneath the eyes appears to have arisen from a real deposit of coloring matter in the pigment-cells of the skin. The ointment of the iodide of potash stimulated the absorbents, causing them to take up and convey away this pigment.

January 20, 1852.

